# Triggers, Bullets and Targets, Puzzle of Membranous Nephropathy

**DOI:** 10.5812/numonthly.2330

**Published:** 2012-09-24

**Authors:** Mohammadreza Ardalan

**Affiliations:** 1 Nephrology Department, Tabriz University of Medical sciences, Tabriz, IR Iran; 2 Mario NegriInstitute of Pharmacological Research, Bergamo, Italy

**Keywords:** Glomerulonephritis, Membranous, Phospholipase A2, Rituximab, 6B1 IgG4 Monoclonal Antibody

## Abstract

**Introduction:**

Idiopathic Membranous Nephropathy (MN) is a common cause of adult nephrotic syndrome. Recently, M-type phospholipase A2 receptor (PLA2-R) has been discovered as the main podocyte antigen in the pathogenesis of idiopathic MN.

**Materials and Methods:**

In this mini review, the author searched English-language MEDLINE for the terms “membranous nephropathy”, “rituximab”, and “phospholipase A2 receptor” up to October 2011.

**Results:**

In support of its earlier discovery, reports from China and Europe confirmed the major pathogenic role of non-complement fixing IgG4 antibody against PLA2-R in the pathogenesis of idiopathic MN. Antibodies against aldose reductase (AR) and manganese superoxide dismutase (SOD2 ), and sub-epithelial deposition of cationic bovine serum albumin (BSA) are also reported in rare occasions. It seems that Rituximab is a good therapeutic choice for those patients who need immunosuppressive therapy.

**Conclusions:**

Great discoveries in the diagnosis and treatment of idiopathic MN have been performed but pathogenic mechanism and triggers for anti-PLA2-R production are still unknown.

## 1. Introduction

Idiopathic Membranous Nephropathy (iMN) is a common cause of adult nephrotic syndrome. For more than 50 years researchers have debated for an autoimmune basis of MN, but the auto-antigen remained elusive (1). In 2009, Beck and colleagues discovered that autoantigen is mainly a M-type trans-membrane phospholipase A2 receptor (PLA2-R) located on podocytes, and autoantibody is mainly a non-complement fixing- IgG4 (1). With these new findings iMN should no longer be considered as an idiopathic disease. Very recently a new indirect immunofluorescence test (IIFT) enabledeasy detection of anti-PLA 2 R antibody in the serum of patients with iMNby which anti-PLA 2 R antibodies were found in 52% of patients with biopsy-proven iMN (2). However, the triggers of autoantibody production and mechanisms of its action are yet unknown and investigations for other likely antigens are in progress (3, 4).

## 2. Materials and Methods

Inthis mini review, the author searched the MEDLINE as at initiation of ideas about Rituximab treatment and M-type phospholipase A2 receptor autoantibody in iMN up to October 2011. All English-language studies that reported diagnosis and treatment of idiopathic MN were searched using the terms “membranous nephropathy”, “rituximab” and, “phospholipase A2 receptor”. We included important studies by cross-referencing. We also included author’s definitions of some conditions such as complete remission (CR) and partial remission (PR) in our review.

## 3. Results and Discussion

### 3.1. Anti-Phospholipase A2 -Receptor Antibodies

In its early discovery by Beck and colleagues, using Western blot assay 26 out of 37 (70%) serum samples of American patients with iMN showedan antibody (mainly IgG4) against a 185-kD PLA2-R glycoprotein in the glomerular extract (1). In a report from China, by the use of a Western blot assay, 49 out of 60 patients (82%) with idiopathic MN demonstrated detectable anti-PLA2-R auto-antibodies (5). In an European cohort of iMN patients, they measured anti-PLA-R auto-antibody levels by a Western blot immunoassay in serum samples of patients in nephritic proteinuria, remission, or relapse periods. Fourteen out of 18 (77.8%) patients showed IgG4 auto-antibody in their active phases; it was decreased significantly during remission and increased again during relapse (6). In a recent report, PLA2-R autoantibody was found in5 out of 10 patients with recurrent iMN but in none of 9 patients with de novoMN following renal transplantation (7).

### 3.2. Antigen Targets

The super-family of phospholipase A2 (PLA2) consists of distinct types of structurally related enzymes (8). Family of secreted PLA2s (sPLA2) by itself has a widespread distribution in nature and presents in different fluids and tissues as an antibacterial protection (9, 10). PLA2 -IIA is found in snake venom and other serpent’s maxillary glands (11). High-molecular weight cytosolic PLA2 (cPLA2) is located on the cell membrane or endoplasmic reticulum and acts as intracellular second messenger. There is an intensive relationship between cPLA2 and sPLA2 . PLA2-R isexpressed not only in human kidney but also in lung, pancreas, placenta, and skeletal muscle (13). Inflammatory cytokines such as IL-6, TNF-α, and IL-1β induce the synthesis and release of sPLA2 from different cells (13). Mammalian sPLA2 could attach to PLA2-R and induce pro-inflammatory signals by a receptor-mediated mechanism. Heymannnephritis (HN) is an experimental rat model of Nephrotic syndrome (NS), but Megalin, the auto-antigen of HN does not express on human podocytes. Epithelial cell injury in Heymann nephritis is induced by complement C5b-9 deposition, and sub-lytic injury to podocytes, activate finally cPLA2, an important mediator of podocyte injury and actin cytoskeleton collapse, through increasing intracellular calcium and protein kinase C (PKC) activation, and extracellular signal–regulated kinase (ERK) (3, 14). IgG4 is unique among IgG subclasses because it weakly activates the complement. Whether IgG4–PLA2–R interaction in human is similar to sub-lytic C5b-9 induced podocyte injury in Heymann nephritis and what the triggers are for anti- PLA2-R production still remainedunknown.

**Figure 1:**
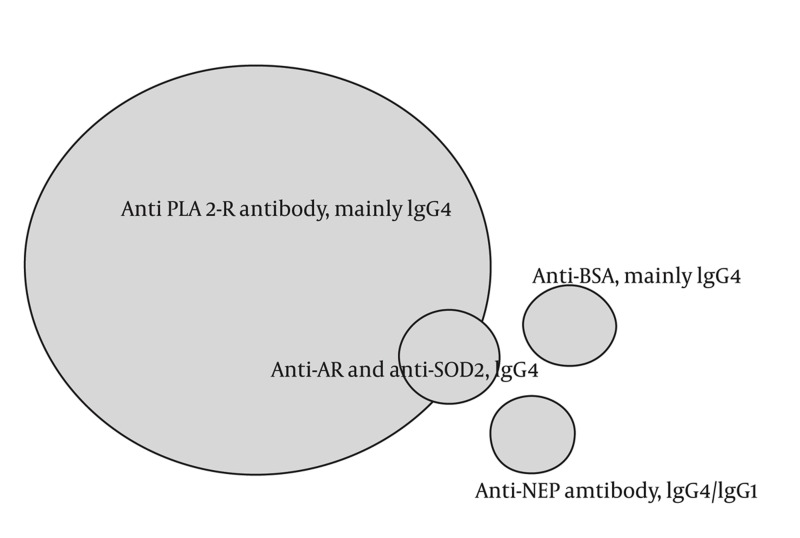
IgG4 Antibody Against M-type Phospholipase A2 Receptor (PLA-R) Is the Main Antibody in Pathogenesis of Idiopathic Membranous Nephropathy. Antialdosereductase (AR) and manganese superoxide dismutase (SOD2) antibodies may work as the second hit after primary anti PLA2-R antibody injury. Anti neutralendopeptidase (NEP) hasbeen reported in rare instances of neonatal MN, and antibody against cationic bovine serum albumin(BSA) hasbeen reported in a small group of children with idiopathic MN.

PLA2-R has been discussed as the major candidate antigen of idiopathic MN (Table1 and Figure 1). The nature of antigens involved in remaining cases of idiopathic MN is unclear. In a very rare form of neonatal nephritic syndrome, neutral endopeptidase (NEP) deficient mother may develop antibody against NEP during conception and transmitthe antibody during next pregnancy to subsequent fetus. Transmitted antibody is mainly IgG1. Notably, when the transmitted antibody is non-complement fixing IgG4, membranous nephropathy is not developed in the child (15, 16).

**Table 1:** Target Antigens, Antibodies, and Possible Triggers in Idiopathic MN [Table-fn fn359]

Target Antigen	Antibody	Trigger	Presentation
** NEP ^a^ (Podocyte antigen) **	Anti-NEP antibody both IgG4 and IgG1, disease is not developing if the transmitted antibody from mother is a non-complement fixing IgG4 subclass	NEP deficiency	Neonatal iMN^a^
** PLA2-R ^b^ (Podocyte antigen) **	Anti PLA2-R antibody mainly a non-complement fixing IgG4 antibody	Unknown	iMN
** AR/SOD2 (Cryptic podocyteantigens) **	Anti-AR and anti-SOD2 IgG4 antibody with C5b-9 deposition (6)	These antigens are exposed after primary injury	iMN
** BSA^c^ (Extrinsic, Planted antigen) **	Anti-BSA, mainly IgG4 and IgG1 formation with C5b–C9 Deposition	Undigested BSA enters thecirculation	IMN (Childhood)

Abbreviations: AR, Aldose reductase; BSA, cationic bovine serum albumin; iMN, idiopathic membranous nephropathy; NEP, neutral endopeptidase; PLAR, M-type phospholipase A2 receptor; SOD2, manganese superoxide dismutase.

^a^NEP deficient mothermay develop antibody against NEP during conception.

^b^PLA-R, a trans-membrane protein located on podocytes

^c^Cationic bovine serum albumin binds to anionic glomerular capillary wall.

Antibody against aldose reductase (AR) and manganese superoxide dismutase (SOD2) have been detected in sera and in elutes prepared from renal biopsy tissue of a group of patients with idiopathic MN (17). Superoxide dismutase-2 (SOD2), aldose reductase (AR), and α-enolase are cryptic cytosol proteins. A temporal hierarchy is possible whereby as a consequence of primary podocyte damage by anti-PLA2-R, these cryptic antigens are exposed to immune system (3).

In a small group of children with iMN, circulating cationic bovine serum albumin (BSA) and glomerular deposits of cationic BSA have been found. Cationic BSA acts as an externally planted antigen. It is pathogenic through binding to the anionic glomerular capillary wall and in-situ formation of immune complexes (IgG4 and IgG1) with sub-epithelial deposition of membrane-attack complex C5b–C9. The amount of intact and undigestedbovine serum albumin entering the circulation is probably higher during infancy and childhood. In this type of iMN, eliminating the bovine milk from the diet could be beneficial (18).

### 3.3. Treatment

Spontaneous remission (not induced by immunosuppressive therapy) is a well-known characteristic of iMN;its incidence ranges from 30% to 50% and carries a favorable outcome. Conservative therapy has been recommended for all patients with iMN and ifproteinuria is decreased more than fifty percent (> 50%) of baseline during the first year of follow-up, it predicts the appearance of spontaneous remission. Immunosuppressive therapy should be considered without delay for patients with deteriorating renal function and for those without proteinuria reduction during this period (19). Therapeutic approaches to iMN mostly relies on steroids and cytotoxics with or without calcineurin inhibitors (20). Rituximab, a monoclonal antibody to CD20 antigen of B cells offers a new therapeutic approach. It safely and persistently reduced proteinuria in a group of patients with iMN who had previously failed to respond to steroids, alkylating agents, or calcineurin inhibitors, or who experienced the relapse after transient remission (21, 22, 23). It is also proposed that rituximab reduces the proteinuria by an immune-independent mechanism by targeting sphingomyelin-phosphodiesterase acid-like3B (SMPDL-3b) protein that preserves the podocyte cytoskeleton (24). Majority of studies gave rituximab at a dose of 375 mg/(sqare meter) of body syrface once weekly for 4 weeks (22, 25, 26). Fervenzaet al. gave 1 g rituximab on days 1 and 15; this regimen was repeated at 6 months if B cells were > 15/µL and proteinuria was still in the nephrotic range (21, 26). In a recent report at the end of two years of follow-up, CR and PR was observed in 16 out of 20 (80%) patients with iMN. More than half of these patients had failed previous immunosuppressive therapy (21). It is believedthat the time has come to conduct a prospective randomized controlled trial in multiple centers with diverse patient populations to assess the efficacy and long-term benefits of rituximab to be compared with traditional immunosuppressive treatment (27).

## 4. Conclusions

Great discoveries have been performed and great questions remain to be answered. Up to now pathogenic mechanism and triggers for anti-PLA2-R production arestill unknown. Idiopathic MN might be exhibited as a heterogeneous entity rather than a uniform disease. Autoimmune process could also target other poorly understood glomerular antigens beyond what have been discovered recently. Latest findings about the pathogenic role of cationic BSA raise the possibility that other food antigens might be involved in development of idiopathic membranous nephropathy.
